# Longitudinal Deformation-Based Morphometry Reveals Spatio-Temporal Dynamics of Brain Volume Changes in Patients with Corticobasal Syndrome

**DOI:** 10.1371/journal.pone.0041873

**Published:** 2012-07-27

**Authors:** Martin Südmeyer, Peter Pieperhoff, Stefano Ferrea, Holger Krause, Stefan Jun Groiss, Saskia Elben, Lars Wojtecki, Karl Zilles, Katrin Amunts, Alfons Schnitzler

**Affiliations:** 1 Department of Neurology, Center for Movement Disorders and Neuromodulation, Medical Faculty, Heinrich Heine University, Düsseldorf, Germany; 2 Institute of Clinical Neuroscience and Medical Psychology, Medical Faculty, Heinrich Heine University, Düsseldorf, Germany; 3 Institute of Neuroscience and Medicine (INM-1, INM-2), Research Center Jülich, Jülich, Germany; 4 C. and O. Vogt Institute for Brain Research, Heinrich Heine University, Düsseldorf, Germany; 5 Department of Psychiatry, Psychotherapy, and Psychosomatics, RWTH Aachen University, Aachen, Germany; 6 JARA – Translational Brain Medicine, Jülich, Germany; University Hospital La Paz, Spain

## Abstract

**Background/Objective:**

Corticobasal syndrome (CBS) is a rare neurodegenerative disorder characterized by a progressive and asymmetric manifestation of cortical and basal-ganglia symptoms of different origin. The spatio-temporal dynamics of cerebral atrophy in CBS is barely known. This study aimed to longitudinally quantify the individual dynamics of brain volume changes in patients with CBS as compared to healthy controls.

**Methods:**

We used deformation-field-based morphometry (DFM) to study volumetric changes of each individual brain in short intervals of a few months. DFM enabled the quantitative analysis of local volume changes without predefining regions of interest in MR images of 6 patients with CBS and 11 matched healthy controls. A total of 64 whole brain 3D-MR-scans were acquired two to eight times over periods of 14 to 26 months. Based on repeated registrations of MR observations to the initial scan, maps of local volume ratio changes were computed.

**Results:**

Compared to controls patients showed significant and increasing volume loss over time in premotor and primary-motor-cortices, somatosensory area 3a, superior parietal areas BA 5/7, and corticospinal tract. Furthermore, significant and asymmetric atrophy was identified in the caudate nucleus head, putamen, pallidum, motor-thalamus and substantia nigra. Temporal lobe was affected in those patients who presented progressive cognitive impairment.

**Conclusions:**

The analysis revealed localized, pathological changes in brains of patients with CBS, which differed significantly from those occurring during aging in healthy controls. As compared to age- and sex-matched controls, brains of CBS patients showed a common degenerating neural network comprising the motor circuit with basal ganglia and motor thalamic nuclei as well as the premotor and primary-motor-cortex.

## Introduction

Although structural changes have been reported in patients with corticobasal syndrome (CBS) using neuroimaging [1], [2], [3], [4], the individual dynamics of progression of clinical symptoms in CBS and their relationship to the topography of structural changes that segregate the neurodegenerative effects are not well understood. In particular at the beginning of the disease local structural changes are indiscernible from those observed during normal aging due to considerable intersubject variability. The currently available tools for the analysis of MR images mainly lack the necessary sensitivity for detecting small changes that occur over a relatively short period of time, such as few months. However, in CBS and other neurodegenerative diseases such tools are desperately needed for an early clinical diagnosis and for the identification of treatment strategies that may modify the disease progression [5].

CBS is a rapidly progressing disease, which comprises different entities including corticobasal degeneration (CBS-CBD), Alzheimer disease (CBS-AD), progressive supranuclear palsy (CBS-PSP), frontotemporal lobar degeneration (FTLD), multisystem tauopathy, and parkinsonism linked to chromosome 17, wherefore pathological diagnosis is required as gold standard [6], [7], [8]. The nomenclature and included subtypes, however, are still a matter of discussion [9].

In 24–55% of all patients presenting with symptoms of CBS the underlying pathology is CBS-CBD [7], [10]. CBS-CBD commences between the age of 60–80 with typical signs of a progressive, asymmetric, akinetic-rigid syndrome including the clinical hallmarks of an ideomotor limb apraxia, alien limb syndrome and cortical sensory loss that respond poorly to levodopa therapy [11]. In addition, CBS-CBD can lead to frontotemporal dementia and non-fluent aphasia [10]. Due to this clinical heterogeneity, an overlap with other neurodegenerative diseases is common and may lead to clinical misdiagnosis [9]. In patients with clinical diagnosis of CBS-CBD, only 50% revealed pathologically proven CBD, and PSP was mainly detected in false positive cases [7], [10]. Furthermore, unusual types of Alzheimer’s disease with prominent motor features or FTLD may have a similar clinical presentation [7], [8], [9], [10].

The major histopathological feature of CBS-CBD is a widespread accumulation of abnormal phosphorylated tau protein in both glial cells and neurons, astrocytic plaques, and coiled bodies with gliosis of the substantia nigra [12]. Focal cortical neuronal loss and underlying white matter degeneration is primarily present in sensory and motor areas of the parietal and frontal cortex with relative sparing of temporal as well as occipital regions [11]. However, the localization of these findings overlaps with other tauopathies that may explain clinical similarity, leading to an ongoing discussion if these diseases in fact reflect separate entities [8], [9].

During the last years different functional and morphometric in-vivo studies (primarily cross-sectional) focused on the subtype differentiation of CBS [3], [4], [13]. Recently, Whitwell and colleagues published cross-sectional imaging findings of autopsy-proven CBS patients [4]. According to the pathological diagnosis, an increased focal cortical atrophy in the premotor und supplementary motor area was due to CBS-CBD and CBS-PSP pathology, whereas FTLD revealed a stronger frontotemporal and CBS-AD a more prominent temporo-parietal pattern of atrophy.

We here applied magnetic resonance imaging (MRI) and deformation field morphometry (DFM) in six clinically diagnosed CBS patients and eleven age- and sex-matched healthy controls from an ongoing follow-up study employing a longitudinal design. DFM is capable of detecting even subtle regional volume changes in the entire brain in small samples. The topography of significant volume changes was identified using probabilistic maps of cytoarchitectonic areas [14], [15], [16].

**Table 1:** Demographics and clinical findings of CBS patients.

	Case 1	Case 2	Case 3	Case 4	Case 5	Case 6
Gender	f	m	m	f	f	m
Age, y	72	69	76	68	62	65
Education, years	<12	<12	<12	>12	>12	<12
Disease duration, years	1	2	2	2	3	4
Clinical asymmetry	ri > le	le > ri	le > ri	le > ri	ri > le	ri > le
Diagnostic MRI (T initial)	normal	normal	atrophic	atrophic	normal	atrophic
MRI acquisition (Tesla)	1.5	3.0	3.0	3.0	3.0	1.5
Follow-up, months	26	25	24	14	16	19
*Clinical signs (T initial/final)*						
Rigidity	+/+	+/+	+/+	+/+	+/+	+/+
Bradykinesia	+/+	+/+	+/+	+/+	+/+	+/+
Ideomotor apraxia	+/+	+/+	+/+	+/+	+/+	+/+
Alien limb syndrome	−/+	−/+	−/+	−/−	+/+	+/+
Dystonia	−/+	−/−	+/+	+/+	−/+	+/+
Astereognosia	−/+	−/−	−/+	−/+	−/−	−/+
Myoclonus	−/−	+/+	−/−	−/−	+/+	+/+
Mirror movements	−/+	−/+	+/+	−/−	−/−	−/−
Speech apraxia	−/+	−/+	−/−	−/+	−/−	−/−
Supranuclear palsy	−/−	−/+	−/+	−/+	−/−	−/−
Babinski sign	−/+	−/−	−/−	+/+	−/−	−/−
*Motor testing*						
UPDRS-III (T initial, Off/On)	18/18	22/21	35/33	45/44	21/20	25/25
UPDRS-III (T final, On)	33	46	58	64	45	32
FT values (T initial, ri/le)	3/42	49/38	32/12	57/0	18/44	1/26
FT values (T final, ri/le)	0/19	0/0	12/0	35/0	8/25	0/7
*Florida Apraxia Score*						
T initial (ri/le)	56/4	2/30	0/14	5/56	37/18	52/33
T final (ri/le)	60/49	38/54	8/28	13/56	42/25	54/51
*Neuropsychological testing*						
MMSE (T initial)	29/30	27/30	22/25	29/30	30/30	25/30
MMSE (T final)	29/30	19/30	21/25	29/30	27/30	26/30
MDRS (T initial)	n.d.	126/144	94/103	142/144	144/144	n.d.
MDRS (T final)	133/144	96/144	90/103	143/144	131/144	107/144

Abbreviations: MMSE  =  Mini Mental State Examination, MDRS  =  Mattis Dementia Rating Scale, MRI  =  Magnetic resonance imaging, n.d.  =  not done, UPDRS-III  =  Unified Parkinson’s Disease Rating Scale part III, FT  =  Finger tapping, le  =  left, ri  =  right, f  =  female, m  =  male, T initial  =  initial examination, T final  =  final examination, −  =  absent, +  =  present.

## Methods

A sample of six patients with the clinical diagnosis of CBS attending the Movement Disorder Centre of the University Hospital Düsseldorf was prospectively recruited. At the time point of study inclusion (T initial) all patients underwent detailed neurological examination (including testing of the autonomic nervous system and formal psychometry using Florida Apraxia Screening Test (FAST) [17], Mattis Dementia Rating Scale (MDRS) [18], Mini Mental State Examination (MMSE) [19]), routine laboratory tests, and volumetric as well as diagnostic magnetic resonance brain imaging to exclude symptomatic parkinsonism during hospitalization over a time period of 5–8 days. Additionally, the Unified Parkinson Disease Rating Scale part III (UPDRS-III) was determined 12 hours after discontinuation of antiparkinsonian medication and after oral administration of 250 mg L-Dopa [20]. Furthermore, one minute of finger tapping (FT) was performed with the left and right forefinger; subjects were required to alternatingly press specific counting devices which were fixed on a board as rapidly as possible. In addition, all patients underwent clinical and MRI follow-up examinations performed on the same day during the course of 20.7±5 months (4–8 serial MRIs). In most cases, intervals between consecutive scans were about three to four months. Final diagnosis of CBS was made in a consensus panel arrangement by two movement disorder specialists (A.S., M.S.), taking into consideration data of carefully assessed longitudinal neurologic follow-up examinations, possible response to dopaminergic treatment, diagnostic MRI and published clinical criteria [6], [21], [22]. No subjects were excluded from the study, but two follow-up scans were discarded from further analysis due to movement artefacts.

**Table 2:** Local volume ratio (LVR) changes (Median ± SEM) as % for different cortical and subcortical regions of 11 healthy controls and 6 CBS patients at final follow-up examination (T final) as compared to the initial examination (T initial).

		Controls	CBS	P value	Controls	CBS	P value
		contralateral	contralateral		ipsilateral	ipsilateral	
*Global Hemisphere*		−0.22±0.13	−1.25±0.38	0.18	−0.36±0.12	−1.09±0.37	0.22
*Frontal cortex*							
area 4	c	0.03±0.20	−3.07±1.92	0.0002*	−0.36±0.19	−3.36±1.04	0.0002*
area 6	c	−0.25±0.22	−3.69±1.94	0.0002*	−0.12±0.20	−3.53±1.29	0.0002*
superior frontal gyrus	m	−0.21±0.22	−0.80±0.58	0.40	−0.41±0.18	−1.40±0.78	0.30
medial frontal gyrus	m	−0.12±0.27	−0.49±0.51	0.40	−0.47±0.15	−1.18±0.71	0.03
inferior frontal gyrus	m	−0.17±0.24	0.59±0.36	0.35	−0.34±0.18	−0.82±0.63	0.52
orbitofrontal cortex (10,11,47)	m	−0.16±1.32	1.85±1.79	0.06	−0.52±0.79	−0.55±0.91	0.65
gyrus rectus	m	−0.62±0.30	1.22±0.77	0.05	−0.13±0.32	0.99±0.65	0.08
Broca’s area (44/45)	c	−0.28±0.27	−0.05±0.41	0.73	−0.30±0.19	−1.24±0.66	0.22
*Parietal cortex*							
area 3a	c	−0.46±0.28	−3.21±1.10	0.01*	−0.54±0.27	−2.79±0.80	0.01*
area 3b	c	−0.03±0.18	−1.96±0.98	0.09	0.06±0.18	−1.46±0.74	0.22
area 1	c	0.27±0.31	−1.77±0.74	0.05	0.12±0.25	−1.43±0.61	0.08
area 2	c	0.02±0.24	−1.78±0.81	0.03	−0.02±0.24	−0.61±0.78	0.40
superior parietal lobule (5,7)	c	−0.10±0.20	−2.62±0.43	0.001*	−0.18±0.19	−1.47±0.66	0.007*
Precuneus	c	−0.06±0.28	−0.81±0.37	0.15	0.21±0.31	−0.75±0.70	0.18
inferior parietal lobule	c	0.26±0.20	−0.93±0.67	0.52	−0.19±0.18	−0.15±0.56	0.96
*Occipital cortex*							
primary visual cortex	c	−0.04±0.16	0.21±0.51	0.59	−0.23±0.12	0.23±0.40	0.22
secondary visual cortex	c	−0.29±0.17	−0.26±0.54	0.81	−0.17±0.14	−0.51±0.53	0.52
lingual gyrus	m	−0.19±0.19	0.27±0.48	0.59	−0.08±0.13	0.14±0.48	0.66
Cuneus	m	−0.23±0.18	0.33±0.47	0.59	0.31±0.20	0.05±0.45	0.73
*Temporal cortex*							
superior temporal gyrus	m	−0.36±0.14	0.46±0.95	0.40	−0.38±0.16	0.42±0.46	0.18
medial temporal gyrus	m	−0.45±0.19	0.11±0.90	0.66	−0.47±0.23	−0.40±0.85	0.73
inferior temporal gyrus	m	−0.13±0.18	−0.12±1.17	1.00	−0.30±0.18	−0.27±0.90	0.88
Amygdale	c	−0.18±0.25	0.46±0.95	0.59	−0.45±0.23	0.42±0.46	0.30
entorhinal cortex	c	−0.14±0.31	−1.22±0.82	0.18	0.14±0.32	−1.27±0.89	0.40
fusiform gyrus	m	−0.21±0.15	−0.24±0.76	1.00	−0.51±0.18	−0.79±0.66	0.22
parahippocampal gyrus	m	−0.15±0.34	0.04±1.05	0.88	0.20±0.35	1.09±1.00	0.26
TE3 (Wernicke)	c	0.14±0.26	0.82±0.94	0.59	−0.73±0.39	0.23±0.80	0.30
*Basal ganglia*							
caudate nucleus	m	−0.61±0.25	−0.31±0.45	0.96	−0.43±0.19	2.04±0.85	0.05
head of caudate nucleus	m	−0.47±0.21	−2.76±0.41	0.0006*	−0.09±0.23	−1.67±0.81	0.30
Putamen	m	−0.73±0.19	−3.84±0.76	0.0001*	−0.56±0.23	−2.69±0.93	0.001*
Pallidum	m	−0.86±0.21	−3.83±0.91	0.0006*	−0.40±0.26	−3.76±1.14	0.01*
*Thalamus*							
central medial nuc.	c	−1.84±0.30	−2.24±0.42	0.46	−0.95±0.41	−2.50±0.53	0.03
dorsomedial nuc.	c	−1.16±0.59	−2.76±0.59	0.35	−0.72±0.38	−2.32±1.46	0.18
lateral posteriordorsal nuc.	c	−1.00±0.33	0.85±0.42	0.18	−1.04±0.28	0.76±0.25	0.15
anterior nuclei	c	−0.69±0.46	0.11±0.84	0.29	–0.71±0.35	0.23±0.58	0.39
ventral anterior nuc.	c	−0.79±0.22	−3.06±0.68	0.0003*	−0.52±0.27	−4.42±0.88	0.003*
ventral lateral nucl.	c	−0.47±0.20	−3.67±0.70	0.0001*	−0.65±0.26	−2.28±0.91	0.05
ventral posterior nuc.	c	−0.97±0.29	−1.55±0.87	0.46	−0.33±0.19	−1.69±0.80	0.46
pulvinar	c	−1.54±0.30	0.17±1.55	0.15	−1.04±0.18	0.39±0.90	0.05
*Midbrain*							
nucleus ruber	c	−0.28±0.27	−1.25±0.67	0.03	−0.90±0.30	−2.34±0.96	0.15
substantia nigra	m	−0.44±0.25	−2.58±1.15	0.001*	−0.13±0.33	−4.09±1.06	0.007*
*Cerebellum*							
anterior lobe	m	−0.02±0.23	−0.64±0.68	0.66	−0.25±0.25	−0.82±0.72	0.52
posterior lobe	m	0.06±0.20	−0.24±0.46	0.81	−0.12±0.16	−0.50±0.46	0.88
*Ventricles*	m	3.39±1.43	18.75±4.84	0.007*	1.67±0.66	17.49±4.08	0.002*
*Corticospinal tract*	f	−0.57±0.19	−4.30±1.21	0.0002*	−0.34±0.17	−3.92±0.85	0.0003*

P values are given for a two-sided Mann-Whitney-U-test. *indicates significant P values (corrected for multiple comparisons by controlling the false discovery rate at 0.05). F indicates myeloarchitectonically defined maps of fibers, M indicates macroanatomically and C cytoarchitectonically defined areas.

The control group consisted of eleven age- and sex-matched, right-handed healthy volunteers with no previous history of neurological or medical disorder, who underwent 21.4±2.7 months of follow-up with 2–4 serial MRIs obtained (Table S1).

**Figure 1:**
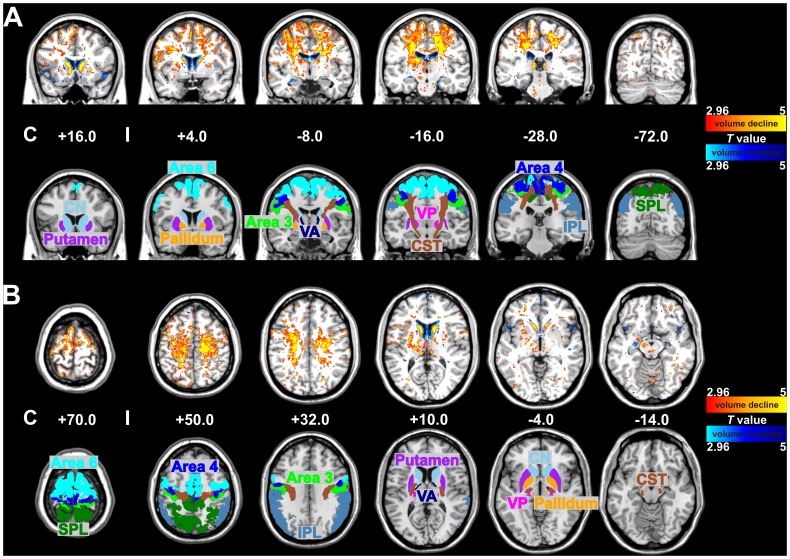
Statistical maps (A: coronal and B: axial sections) of voxel-wise volume differences (upper trace) in comparison to probabilistic maps of cytoarchitectonically defined areas (lower trace) between 6 CBS patients and 11 age- and sex-matched controls (P<0.01). The colored bar represents the *T* score of significant voxels (red to yellow indicate volume decline, blue indicate volume increase). Abbreviations: IPL  =  Inferior Parietal Lobule, CN  =  Caudate Nucleus, CST  =  Corticospinal Tract, SPL  =  Superior Parietal Lobule, VP  =  Ventral Posterior Nucleus of the thalamus, VA  =  Ventral Anterior Nucleus of the thalamus, C  =  Contralateral to the clinically most affected side, I  =  Ipsilateral to the clinically most affected side.

### Ethics

Patients examined in this study were recruited from a large longitudinal study on patients with movement disorders, approved by the local ethics committee (study-no. 2849, “Ethikkommission der Medizinischen Fakultät der Heinrich-Heine-Universität Düsseldorf”, http://medfak.uni-duesseldorf.de/dekanat/kommissionen/ethikkommission/, Ethikkommission@med.uni-duesseldorf.de). The procedure and the reason for this examination in particular were explained to all patients and carers as well as healthy controls in full detail. At study inclusion all patients had adequate capacity/ability to personally give written informed consent as approved by the detailed neurological and neuropsychological testing. The study is in accordance with the Declaration of Helsinki.

**Figure 2:**
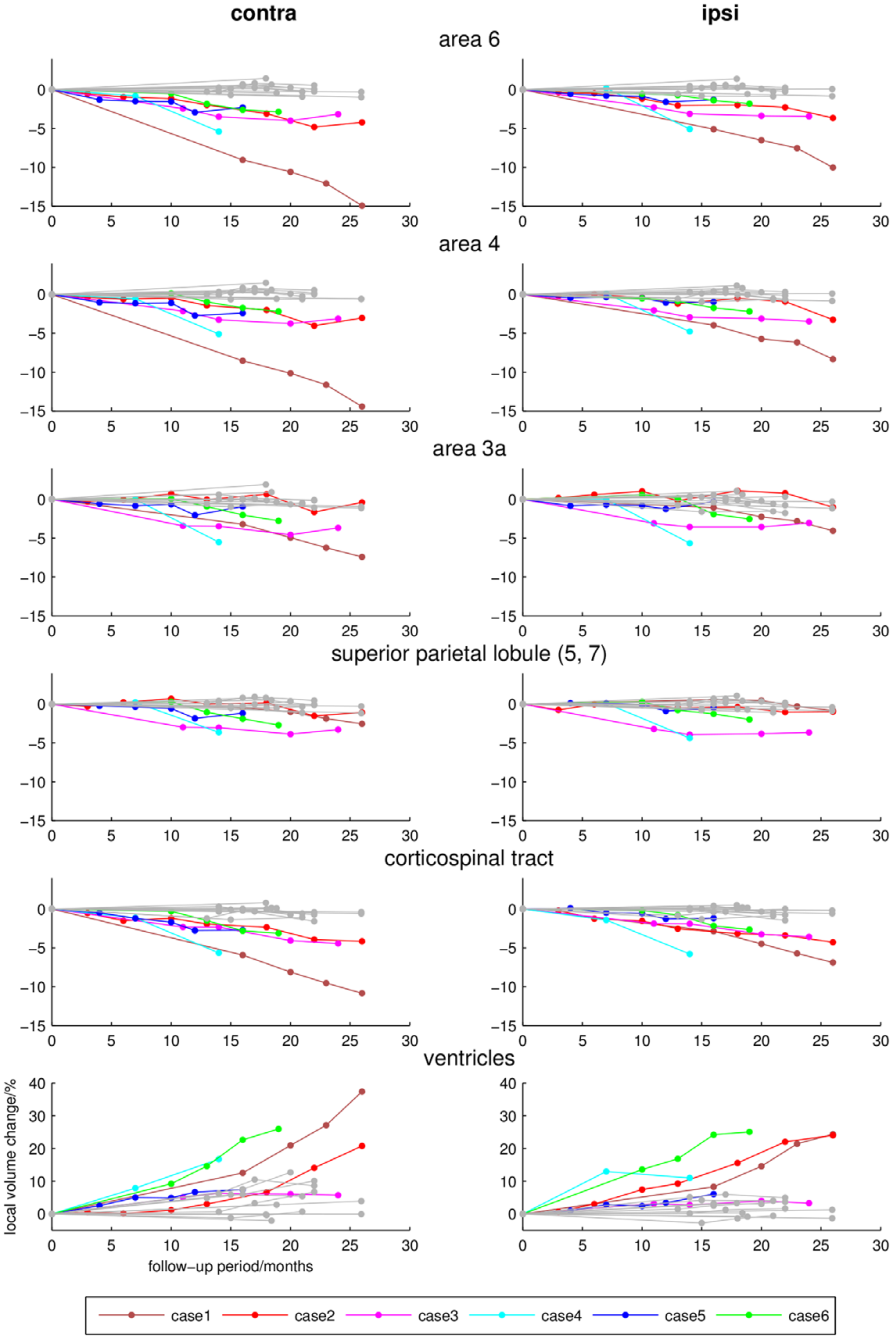
Longitudinal local volume ratios (percent, y-axis) during follow-up examination (months, x-axis) of different cortical regions, the corticospinal tract and lateral ventricles that revealed significant group differences between CBS patients (coloured lines) and controls (grey lines). Note that the y-axis of the lowermost graph is differently scaled.

### MRI

Brains of the patients and the healthy controls were scanned in a 1.5 Tesla (Magnetom Vision, Siemens Medical Solutions; Erlangen, Germany) or 3 Tesla (Magnetom Trio Tim System, Siemens Medical Solutions; Erlangen, Germany) MR-tomograph with a standard CP head coil. Pulse sequences were as follows: sagittal 3D gradient echo sequence (repetition time (TR) 9.7 ms, echo time (TE) 4 ms, 1 mm slice thickness for 1.5 Tesla and TR 2300 ms, TE 2.98 ms, 1 mm slice thickness for 3 Tesla, with one signal acquired), field of view (FOV) 256-mm and 256×256 matrix. All follow-up scans of a single patient or control were acquired by using the same scanner as for the initial scan (Table 1, Table S1).

**Figure 3:**
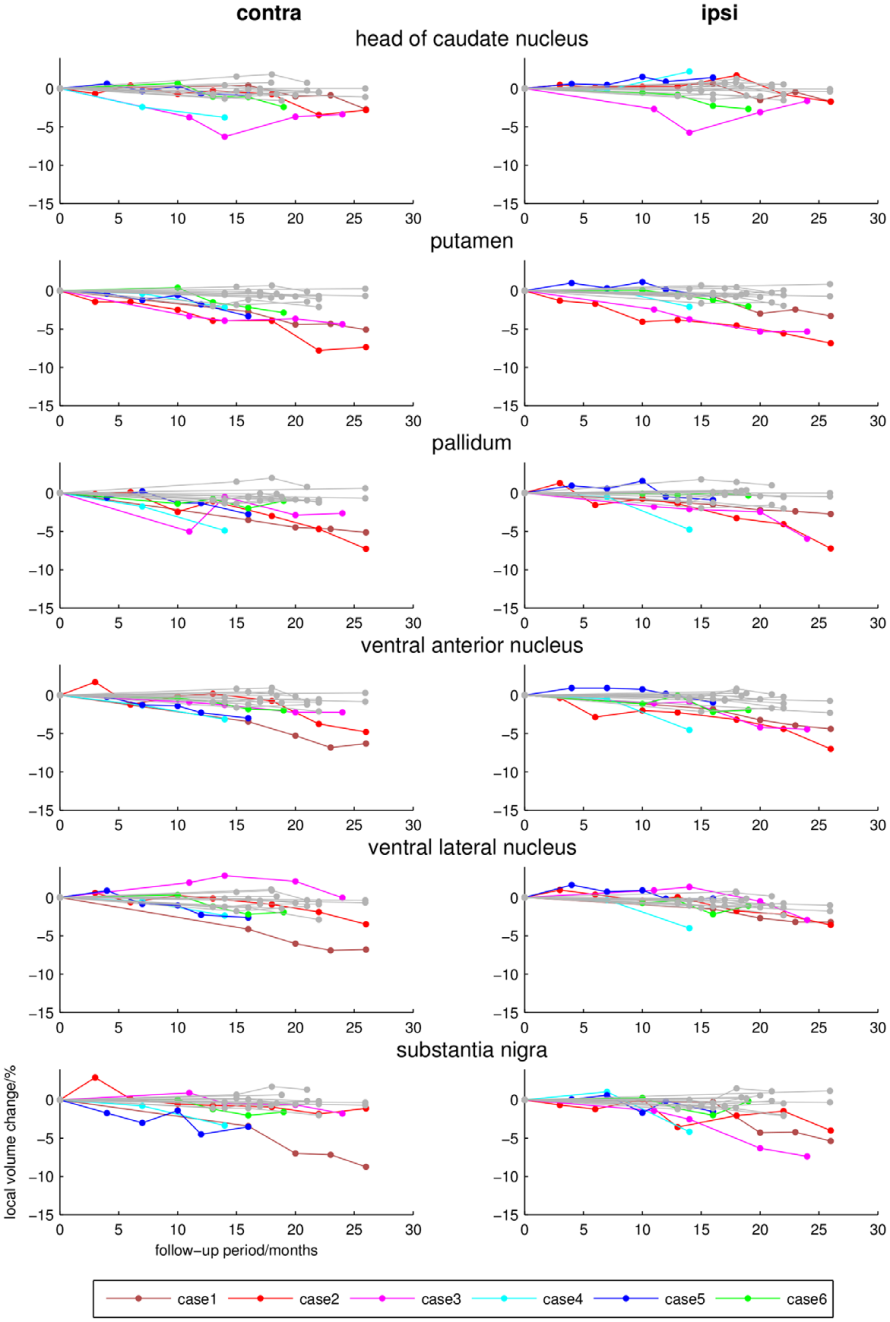
Longitudinal local volume ratios (percent, y-axis) during follow-up examination (months, x-axis) of different subcortical regions that revealed significant group differences between CBS patients (coloured lines) and controls (grey lines).

### Deformation Field Morphometry (DFM)

A detailed description of the applied morphometric technique has been published [16]. Hence, we only provide a brief outline here. Serial MR images of each subject were examined for intraindividual differences between each follow-up and the initial MR image (Ti). Therefore, the follow-up images were registered to the initial image using a non-linear registration. As a result of registration, deformation fields were calculated for each subject and each follow-up imaging. The deformation fields were transformed to “local volume ratio” (LVR) maps [16]. The LVR data indicate voxel-wise volume differences of the brain between the initial and corresponding follow-up MR observations. Therefore, a sequence of each subject’s LVR maps encodes structural changes in each voxel of the brain.

**Figure 4:**
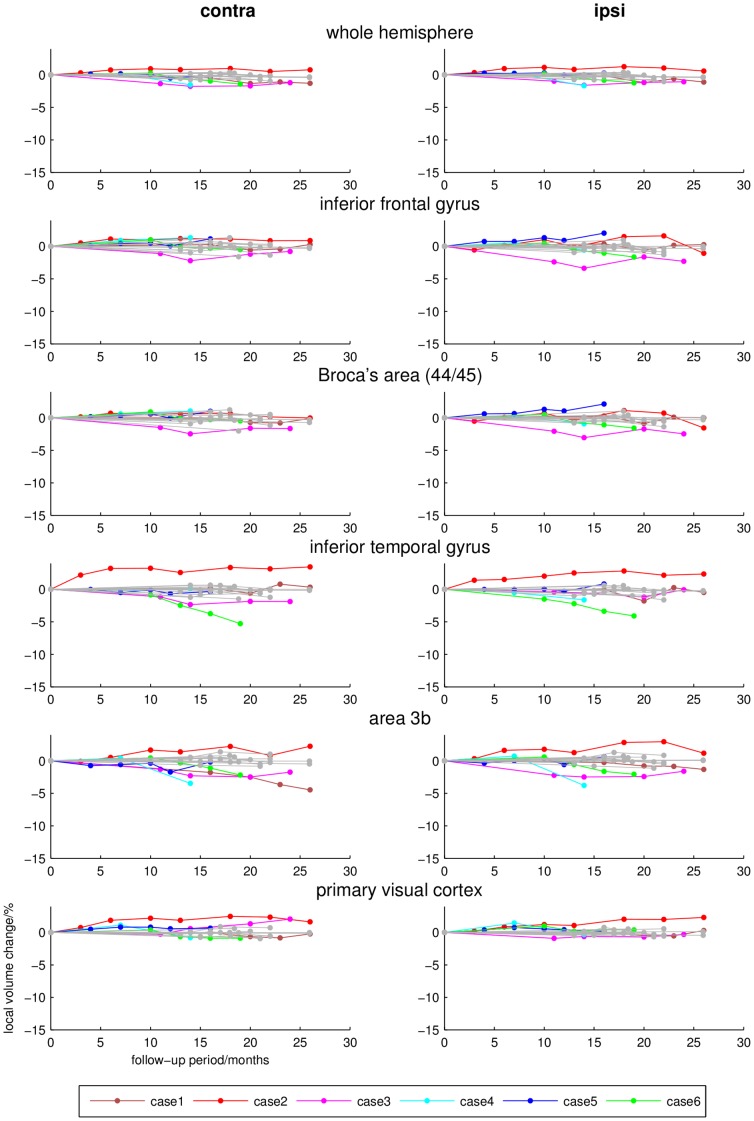
Longitudinal local volume ratios (percent, y-axis) during follow-up examination (months, x-axis) of different cortical and subcortical regions that revealed no significant group differences between CBS patients (coloured lines) and controls (grey lines). Note that at individual level in cases 3 and 6 a mild affection of the inferior temporal gyrus is evident.

### Analysis of Anatomical Brain Regions

LVR maps were analysed in two different ways [16]: (a) Voxel-wise analyses; maps showing individual changes of brain structure in the native space of each subject were generated, as well as statistical maps showing differences on the group level. The latter analysis required all subjects’ LVR-maps of the last examination to be transformed into the standard space of the MNI single-subject template [23]. Differences between the transformed maps were examined by SPM 8 (http://www.fil.ion.ucl.ac.uk/spm). Voxel-wise analyses enable to visualize small regional effects. On the other hand, they are also prone to uncertainties due to image registration, which may impact voxel-wise statistical analysis. (b) Volume changes of anatomical regions; such changes were calculated as the weighted sum of the voxel-wise LVR-values (see below) in regions of interest. Hence the impact of registration errors of single voxels is reduced as compared to a), the robustness of the volume measures is higher. In addition, reducing the volume change data of millions of voxels to a much lower number of regional volume data permits a better visualization. For example, representations of individual time-dependent volume changes as charts provide important information about the individual course of the disease as well as inter-individual variability in an intuitive manner, whereas similar representations by statistical parametric maps are hardly possible.

**Figure 5:**
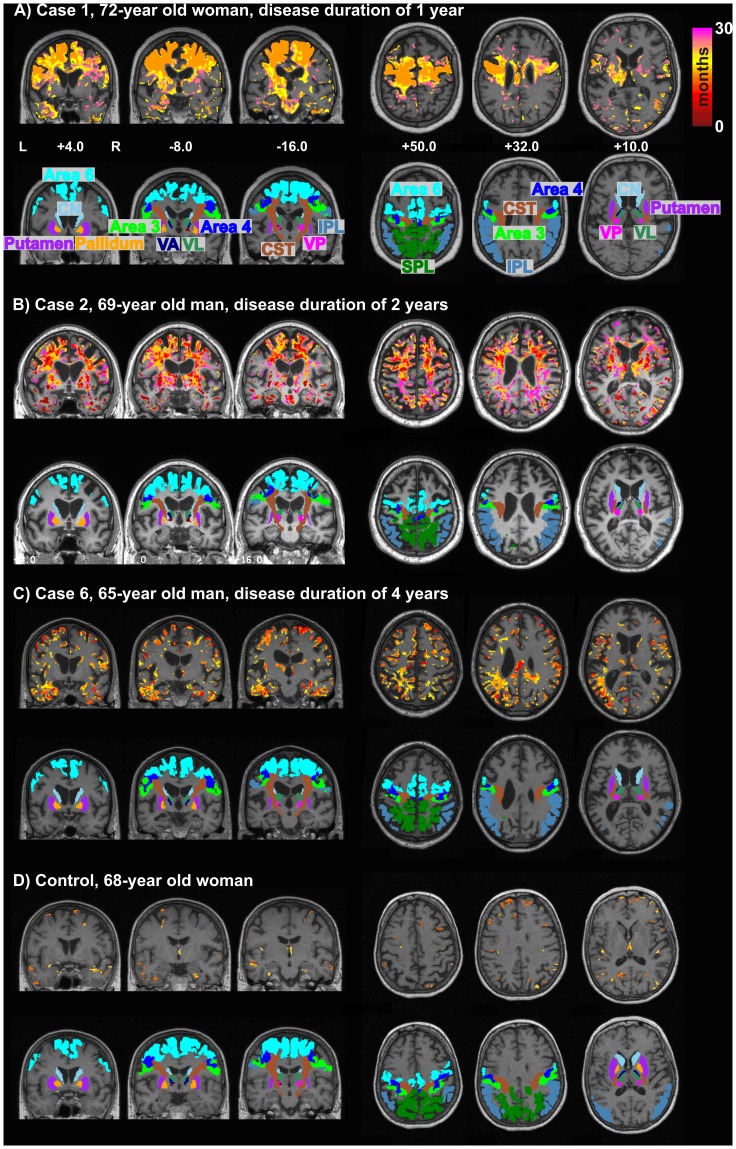
Topography of brain volume changes as detected by DFM analysis in comparison to probabilistic maps of cytoarchitectonically defined areas that were superimposed on each individual MRI-Scan in (A–C) three CBS patients (case 1, 2 and 6) and (D) a healthy control during long-term follow-up examination. The color coding of the voxels indicate the time interval since the initial scan, when their volume had declined by 5% relative to the initial time point. Thus these images show the temporal spreading of the atrophy in each subject’s brain (e.g. red or yellow mean, that after 10 or 20 months, respectively, the voxel volume had shrunk by 5%). Note that in (A) patient 1 and (B) patient 2 showed a more prominent volume decline within area 6 and basal ganglia nuclei, whereas (C) patient 6 revealed lesser volume changes with stronger involvement of temporal regions. The control brain (D) showed only minimal brain volume alterations. Abbreviations: CN  =  Caudate Nucleus, CST  =  Corticospinal Tract, IPL  =  Inferior Parietal Lobule, SPL  =  Superior Parietal Lobule, VA  =  Ventral Anterior Nucleus of the thalamus, VL  =  Ventral Lateral Nucleus of the thalamus, VP  =  Ventral Posterior Nucleus of the thalamus, L  =  Left, R  =  Right.

Probabilistic maps of cytoarchitectonically defined cortical areas and subcortical nuclei, as well as myeloarchitectonically delineated fiber tracts (http://www.fz-juelich.de/inm/inm-1) were used for measurements of localized changes in circumscribed anatomical entities. These structures have been delineated in histological sections of ten post-mortem brains [14], [24], [25], [26]. The probabilistic maps were registered to the T1-weighted single-subject template of the Montreal Neurological Institute (MNI) [23]. In the present study, cytoarchitectonic maps of somatosensory and motor cortex areas as well as the superior parietal lobule were particularly relevant [27], [28], [29], [30]. In addition to the presently available probabilistic cyto- and myeloarchitectonic maps, which do not cover the entire cortex and subcortical nuclei, maps of macroanatomically defined structures of the MNI template (http://www.loni.ucla.edu/ICBM/Downloads/Downloads_ICBMtemplate.shtml) were used whenever necessary. All anatomical maps (macrostructural as well as myelo- and cytoarchitectonic) were warped onto each brain volume [15], [16]. For each case, relative volume changes of cyto- and myeloarchitectonically defined regions of interest were calculated as the sum of voxel-wise LVR-values weighted by the voxel-values of probabilistic cyto- or myeloarchitectonical maps:


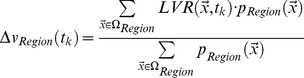


Δ*v_RegionI_(t_k_)*  =  relative volume change of the anatomical region under consideration at time point *t_k_* relative to the initial measurement, *t_k_*  =  time point of a follow-up scan (*k≥*1), *Ω_Region_*  =  set of voxels belonging to the region (at the initial measurement), 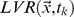 =  local volume ratio in voxel 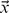 at the follow-up measurement at time point *t_k_*, 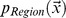  =  probabilistic value of the region in voxel 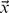.

### Statistical Evaluation

For statistical comparison of the demographic and clinical parameters between CBS patients and control subjects the two-sided t-test was used for normally distributed and the two-sided Mann-Whitney-U-test for non-normally distributed data. Normality of data distribution was assessed using the Kolmogorov-Smirnov test. Differences of *p*<0.05 were considered significant.

Further, we analysed the dynamics of intraindividual volume reduction in myeloarchitectonically defined fiber tracts, cytoarchitectonic areas as well as macroanatomically defined cortical and subcortical areas in CBS patients and controls. Between-group differences of local volume changes at the last follow-up examination were assessed using the two-sided Mann-Whitney-U-test to determine those brain areas, where volume loss was significantly greater in CBS patients than in healthy controls. All controls were right-handed wherefore we defined left-hemispheric data as “contralateral” and right-hemispheric data as “ipsilateral”. This explorative analysis was applied to all brain regions. Testing multiple brain regions in parallel increases the risk of false positive findings. However, the commonly used Bonferroni’s method to control the family wise error will become conservative, in particular when correlations between the tests are present. Such correlations are to be expected, because volume changes of the brain which are caused either by normal aging or by neurodegenerative disease, will in general affect systems of anatomical entities in the brain. Moreover, Bonferroni’s method corrects for the occurrence of *any* false positive result. Therefore, we chose to control the “False discovery rate” at 0.05 in order to correct for multiple comparisons [31], [32]. In addition, the complementary voxel-wise analysis of group differences was calculated in SPM 8.

The gender of the participants and the MR scanner which was used for the image acquisition are two further factors which could intermingle with differences between both patients and controls. The sample size, however, was too low to consider these factors in a multi-way statistical analysis and both groups were balanced against the factor gender (number of males/females: controls 5/6, patients 3/3). Therefore, a gender-related confounding effect was not expected. The effect of the different MR scanner type was analyzed by calculating a t-test of differences between the 1.5-Tesla- and 3.0-Tesla scans within the control group, which did not reach significance. Therefore, both factors were not further considered in the following analyses. Moreover, it should be stressed, that each participant’s sequence of MR scans was acquired in the same scanner (i.e. there was no mixing of scans from different scanners within the same participant).

Descriptive statistics and basic comparisons were analyzed using the MedCalC software package (MedCalc 11.1, Belgium).

### Clinical Parameters

None of the patients had other neurologic diseases or family history for dementia and parkinsonism. Motor symptoms showed no positive response to levodopa during levodopa-test or continuing dopaminergic therapy.

### Case 1

A 72-year-old woman presented with a 1 year history of progressive rigidity, bradykinesia and ideomotor limb apraxia (IMA) of her right arm at the time of initial volumetric MRI. Neuropsychological evaluation and diagnostic MRI scans were normal. During the 26-months follow-up period (examinations after 16, 20, 23, 26 months) the hypokinetic-rigid syndrome (UPDRS-III 18 → 33), and ideomotor apraxia worsened significantly and additionally affected the left upper extremity. Furthermore, a right-sided cortical sensory loss in terms of a tactile agnosia, speech apraxia, Babinski sign and alien limb syndrome appeared. There were no clinical features of clonus or muscle atrophy. After 26 months of follow-up, she revealed no cognitive impairments besides mild visuoconstruction and attention deficits (MDRS 133/144).

### Case 2

A 69-year-old man complained that over the last 2 years his left upper extremity became clumsy and useless. At initial follow-up examination he revealed a moderate left-dominant hypokinetic-rigid syndrome (UPDRS-III 21) as well as myoclonus and ideomotor apraxia of the left hand. Diagnostic brain MRI scans were normal. Neuropsychological testing detected mild deficits in the subitems attention, initiation-perseveration as well as construction of the MDRS (total score 126/144); memory subscales were normal. During the 25-months follow-up period (examinations after 3, 6, 10, 13, 18, 22, 26 months) the hypokinetic-rigid syndrome dramatically worsened (UPDRS-III 21 → 46) and a left upper alien limb syndrome appeared. Furthermore, ideomotor apraxia became bilateral and a speech apraxia was evident. He developed a vertical supranuclear palsy and postural instability. His abilities on formal psychometric testing deteriorated and MDRS declined to 96, showing worsening of attention, initiation-perseveration and construction as well as onset of memory deficits.

### Case 3

A 76-year-old man progressively developed a stiffness and uselessness of his left arm and leg over the course of 2 two years. At initial examination he presented with a left-dominant moderate hypokinetic-rigid syndrome (UPDRS-III 33). Furthermore, he was affected by a solely left-sided ideomotor apraxia and dystonia. Diagnostic MRI scans demonstrated cortical atrophy pronounced in fronto-parietal regions accompanied by a ventricular widening. The neuropsychological examination was limited since the patient was nearly blind due to a senile macular degeneration. Therefore, only verbal testing was possible and MDRS was reduced to a maximum of 103 and MMSE to a maximum of 25 points. He initially revealed minimal deficits in the domains initiation-perseveration and attention. Memory functions, orientation and conceptualization were normal. During the 24-months follow-up (examinations after 11, 14, 20, 24 months), he developed a severe bilateral but left-dominant akinetic-rigid syndrome (UPDRS-III up to 58) and ideomotor apraxia of all limbs. He became chairbound due to the apractic gait dysfunction. Furthermore, he developed an alien limb syndrome and astereognosia of the left arm as well as a supranuclear palsy. Psychometry revealed worsening of initiation-perseveration and onset of mild memory deficits.

### Case 4

A 68-year-old woman developed a severe akinetic-rigid syndrome (UPDRS-III 44) with dystonic and painful contraction of the left upper and lower extremity over the last 2 years. The left leg became apractic with consecutive gait difficulties. Babinski sign was positive, but there were no clinical findings of weakness, hyperreflexia or fasciculations. Diagnostic MRI scans showed mild to moderate cortical atrophy but detailed neuropsychological testing was normal. After a 14-months follow-up period (examinations after 7, 14 months) she was chairbound due to a gait apraxia. The hypokinetic-rigid syndrome worsened and manifested bilaterally (UPDRS-III 44 → 64). Furthermore, a painful left-dominant dystonia affected all limbs, wherefore initiation of botulinum toxin injection was necessary. Moreover, a speech apraxia and astereognosia developed. She still performed normal on psychometric testing. Two months after the last follow-up examination the patient died of a subacute ileus.

### Case 5

A 62-year-old woman presented with a progressive stiffness, slowness and difficulty using the right arm. At initial examination, she had moderate hypokinetic-rigid syndrome (UPDRS-III 20), ideomotor apraxia, alien limb phenomenon and myoclonus of the right upper limb. Diagnostic MRI scans and detailed neuropsychological testing were normal. During a 16-months follow-up period (examinations after 4, 7, 10, 12, 16 months) the hypokinetic-rigid syndrome rapidly worsened and became bilateral (UPDRS-III up to 45). The ideomotor apraxia as well as the alien limb syndrome further increased and she developed a painful dystonia of the right hand. At final visit, formal psychometry revealed severe constructional apraxia as well as slight initiation-perseveration impairment; memory functions were normal.

### Case 6

A 65-year-old man suffered of a 4 year history of progressive right-dominant upper ideomotor apraxia, dystonia, bradykinesia and rigidity as well as right-sided alien limb syndrome. At the time of first scanning neuropsychological examination revealed reduced verbal fluency due to dysphasia, a moderate dyscalculia and slight deficits of additional cognitive functions. Diagnostic brain MRI demonstrated mild left-sided parietal-frontal atrophy. During the 19-months follow-up significant worsening of right-dominant ideomotor apraxia and hypokinetic-rigid syndrome (UPDRS-III 25 → 32) was evident and a right-sided stereoagnosia developed. Furthermore, pronounced memory impairment and onset of visuoconstructive and attention deficits were obvious.

## Results

### Clinical Findings

The clinical data of the CBS patients are summarized in Table 1. The mean age (68.7±5 years, range 62–76 years) and the mean time of follow-up examinations (20.8±5.2 months, range 14–26 months) were similar to those of the control group (mean age 60.1±10.3 years, range 45–79 years, p = 0.06, Mann-Whitney-U-Test, two-sided; mean follow-up 21.4±2.7, range 18–26 months, p = 0.9, Mann-Whitney-U-Test, two-sided).

During the follow-up period CBS patients showed rapid worsening of clinical symptoms, reflected by a significant increase of the UPDRS-III score (p = 0.015, t test), the FAST score (stronger affected limb p = 0.36, lesser affected limb p = 0.04, t test) and the FT scores (stronger affected limb p = 0.04, lesser affected limb p = 0.004, t test). Asymmetric hypokinetic-rigid syndrome and ideomotor apraxia were present in all CBS patients. The second most frequent clinical findings were limb dystonia and alien limb syndrome in 5/6 patients (83%). Neuropsychological follow-up assessment revealed pathologic findings particularly in the domain of initiation and construction. Mild to moderate memory impairment was present in case 2, 3 and 6.

Neurological and neuropsychological scores of the control subjects remained in normal range during the course of the entire study period (Table S1). They revealed constant UPDRS-III (T initial: 0.9±1.4; T final: 1.1±1.3; p = 0.68, Mann-Whitney-U-Test) and MMSE (T initial: 29.9±0.3, T final: 29.9±0.3) values.

### Group Comparison of Regional Brain Volume Changes between CBS Patients and Controls

Table 2 summarizes the between-group comparison of local volume changes at final follow-up examination (T final) within anatomically defined regions between CBS patients and healthy volunteers. Group comparison identified different cortical and subcortical regions with significant atrophy in CBS patients as compared to controls. Bilaterally, a highly significant volume loss of the premotor area 6 and the primary motor area 4 was detected. Furthermore, the superior parietal lobules of both hemispheres and the somatosensory area 3a demonstrated a significant volume decrease relative to controls. Occipital and temporal regions did not show differences in volume reduction between groups, although in individual patients we found a reduction of volume. With respect to the subcortical regions, significant atrophy was identified in the head of the caudate nucleus, putamen, pallidum and substantia nigra. In addition, highly significant gray matter loss was observed within the motor thalamus comprising the ventral anterior (VA) and ventral lateral (VL) thalamic nuclei. Furthermore, prominent atrophy of the corticospinal tract and widening of the ventricles was found. None of the identified regions revealed significant side-to-side differences.

The complementary voxel-wise analysis of group differences revealed the same pattern (Figure 1): Cortex and underlying white matter of the prefrontal and parietal lobes showed strong volume loss, whereas other parts of the neocortex were spared. Moreover, head of caudate nucleus, globus pallidus and thalamus were strongly affected in the patients group.

### Comparison of Individual Atrophy Patterns Across CBS Patients and Healthy Controls

The individual dynamics of brain volume changes in the regions that revealed significant group differences between CBS patients and healthy controls are demonstrated in Figure 2 and Figure 3; examples of other cytoarchitectonically defined areas are shown in Figure 4.

Longitudinal DFM analysis in the control group showed only minimal alterations of the total brain volume. Only ventricles revealed a mild to moderate increase in total volume after the follow-up period of 18–26 months. In contrast to these minimal brain volume changes, individual CBS patients presented severe and focal atrophy within basal ganglia, thalamus, frontal and parietal areas. Involved regions thereby revealed different degrees of spatio-temporal atrophy contralateral as well as ipsilateral to the clinically most affected side. In all cases, there was a prominent bilateral, progressive volume loss in the premotor and primary motor cortex and the corticospinal tract. Patients with shorter disease durations of 1–2 years at study inclusion (cases 1–4) showed a more severe volume decline than patients with longer disease durations of 3–4 years (case 5–6). The contralateral head of the caudate nucleus, putamen and pallidum were affected in all CBS patients. The ventral anterior nuclei of the motor thalamus were most strongly affected and revealed bilateral involvement in all patients. The rates of volume decline in the substantia nigra and the posterior parietal cortical regions were not as prominent and overlapped between CBS patients and normally aging controls. Atrophy of area 3a was only visible in cases 1, 3, 4 and 6. Interestingly, these patients developed an astereognosia during clinical follow-up examination. The superior parietal lobule revealed only mild rates of atrophy. An affection of the temporal lobe, that did not show significant differences at group comparison, was found in cases 3 and 6 who also presented a cognitive impairment. The topography of brain volume changes during longitudinal DFM analysis and the dynamics of progression are shown in Figure 5.

## Discussion

In this study pathological changes in the individual brains of patients with the clinical diagnosis of CBS were compared to normal aging in healthy controls using longitudinal DFM analysis [16]. During two years of follow-up scans, relatively short time intervals of three to four months revealed the spatio-temporal dynamics of cortical and subcortical atrophy. In all CBS patients, a common degeneration within motor circuits, comprising basal ganglia and motor thalamic nuclei as well as the premotor and primary motor cortex was present. Clinical long-term follow-up examination revealed characteristic progressive, asymmetrical cortical and basal-ganglia features that were unresponsive to levodopa and supported the diagnosis of CBS [6], [21], [22].

To increase the in vivo diagnostic accuracy in CBS several imaging techniques have been proposed to detect neurodegenerative brain volume changes [1], [6]. One methodological approach for the differentiation of CBS during lifetime is given by the visualization of the underlying neurodegeneration using morphometric MRI analysis [3], [4], [13], [33], [34]. In this respect, Whitwell and colleagues only recently showed imaging of atrophy patterns in autopsy proven subtypes of CBS that were of diagnostic value to discriminate CBS-CBD, CBS-PSP, CBS-AD and frontotemporal lobar degeneration with TDP-43 immunoreactivity (CBS-TDP) [4]. They demonstrated a prominent focal gray matter loss of the premotor cortex and supplementary cortex in CBS-CBD and CBS-PSP with sparing of inferior frontal lobe in CBS-PSP. In CBS-AD and CBS-TDP a more widespread and distinctive pattern of gray matter loss was shown, with pronounced frontotemporal volume degeneration suggesting CBS-TDP and temporoparietal deterioration suggesting CBS-AD. Consistent with histopathological findings, degeneration of the premotor and supplementary motor area was evident in all of these different CBS subtypes reflecting a common pattern of atrophy in these regions. However, the analysis had a cross-sectional design and did not identify topography and dynamics of structural changes in individual patients [4].

Therefore, the novel aspect of our study is the use of DFM which allows the robust detection and quantification of individual brain atrophy patterns without predefinition of regions of interest. We were able to show the course of atrophy progression within short time intervals of only a few months. Only one report exists, that used longitudinal voxel-based morphometry analysis in a single, not autopsy proven patient, who initially presented with a nonfluent progressive aphasia and later on developed atypical clinical signs of a CBS [35]. Interestingly, this patient revealed an akinetic-rigid syndrome, dystonia and alien limb phenomenon accompanied by an atrophy spreading from the posterior inferior frontal gyrus to insular regions, thalamus, caudate nucleus and medial frontal lobe. We also found volume reduction in most of these areas. Our results demonstrate highest atrophy rates of up to 15% after 26 months in the premotor region, particularily in the cases with shorter disease duration. This might reflect a phenomenon of slowing neurodegenerative processes in later disease stages which has also been shown by neuropathological and PET studies in Parkinson’s disease [36], [37], [38]. In addition, we found in all CBS patients a distinct and asymmetric volume loss in the substantia nigra, striatum, pallidum, motor thalamic nuclei including anterior ventral and anterior lateral nuclei as well as the primary motor cortex. This affection of a functionally interconnected motor circuit concurs with neuropathological results [12] and also with a deformation tensor imaging study that revealed structural changes within parts of the same network including the motor thalamus, the caudate head, the supplementary motor area and the pre−/postcentral region in CBS patients [2]. Therefore, it seems very likely that an anatomically specific network undergoes degeneration in CBS, which causes the clinical symptoms.

In parallel with cortical and subcortical atrophy we detected a widening of the ventricles. This is in agreement with previously described ventricular volume increase in CBS that ranges from 8.3% in CBS-AD to 16.2% per annum in CBS-CBD [34], and with previous aging studies in healthy subjects [39], [40].

In contrast to these regionally localized volume changes in CBS patients, the DFM analysis of the entire brain with follow-up periods of 18–26 months in healthy controls revealed comparatively weak volume alterations, which correspond to reported changes during normal aging as shown in cross-sectional studies [41].

All of our patients showed major basal ganglia degeneration with affection of nigro-striatal regions. It seems plausible that the akinetic-rigid syndrome, often combined with a limb dystonia, was caused by this damage. Another prominent clinical finding was an ideomotor apraxia of at least one limb in all cases as well as an alien limb phenomenon in 5 out of 6 patients, which previously has been shown to be associated with affection of the premotor area [33], [42]. A further distinct clinical feature in 4 out of 6 patients (case 1, 3, 4, 6) was the manifestation of a cortical sensory loss in terms of an astereognosia of the dominantly affected hand. Interestingly, these patients demonstrated distinct bilateral atrophy in the somatosensory area 3a. The volume reduction was more severe on the contralateral side. Tactile object discrimination depends on the conveyance of multiple afferents from cutaneous and deep receptors in joints and muscles of an exploring hand. Hierarchical somatosensory processing of shape is computed by Brodmann’s areas 3, 1, 2 and parts of the supramarginal gyrus as well as the intraparietal sulcus [43], [44]. Areas 3a and 3b receive input from muscle stretch receptors and cutaneous mechanoreceptors [44]. Therefore, it is plausible that the present results explain the cortical sensory loss in CBS. Communication skills were impaired in 4 patients due to a speech apraxia and dysphasia. However, none of the subjects developed a primary progressive aphasia which correlates with our findings of an unaffected Broca’s area in all of our patients [35]. From human imaging and lesioning-studies it is known that the superior parietal cortex plays an important role in visuospatial and attentional processing [45]. Interestingly, the most common neuropsychological impairments in our patients were visuoconstructive and attention deficits which might be related to the detected atrophy of the area 5 and 7 of the parietal lobe. Three patients further developed mild to moderate memory deficits during clinical follow-up examinations, whereby the cases 3 and 6 displayed commencing temporal lobe atrophy, possibly due to an underlying Alzheimer pathology [4], [46].

One limitation of our study is the missing post-mortem confirmed diagnosis making an accurate discrimination of different nosological entities difficult [6], [7], [8], [9], [10]. However, this is an ongoing follow-up study with the strength that the clinical features are very accurately documented and the course of the illness in the examined patients clearly fits the diagnosis of CBS [6], [7], [10], [11], [21], [22], [47]. Another limitation is that patients were examined with 1.5 or alternatively 3.0 T MR-scanners. However, the two different imaging types were matched between groups. Furthermore, the effect of the different scanners was tested within the control group and no significant influence on the measurement of volume changes was found.

In summary, this study shows that longitudinal DFM is a powerful image analysis tool that supports the investigation of the course of CBS brain pathology in individual patients during short time intervals of only a few months, helping to distinguish their temporal gradient of brain volume changes from that of healthy aging. DFM may therefore become a sensitive biomarker for a more accurate clinical diagnosis even at an early disease stage. Additionally, longitudinal DFM studies could become a helpful structural imaging method to identify agents that slow, stop, or even reverse the disease pathology in CBS and also other neurodegenerative disorders involving the central nervous system.

## Supporting Information

Table S1 **Demographic data of the healthy controls.** Abbreviations: MMSE  =  Mini Mental State Examination, MRI  =  Magnetic resonance imaging, n.d.  =  not done, UPDRS-III  =  Unified Parkinson’s Disease Rating Scale part III, FT  =  Finger tapping, le  =  left, ri  =  right, f  =  female, m  =  male, T initial  =  initial examination, T final  =  final examination.
(DOC)
