# Supplementary material for: Longitudinal Deformation-Based Morphometry Reveals Spatio-Temporal Dynamics of Brain Volume Changes in Patients with Corticobasal Syndrome
Source: PLoS One. 2012 Jul 27;7(7):e41873. doi: 10.1371/journal.pone.0041873 (PMC3407125; doi:10.1371/journal.pone.0041873)
Supplement: Table S1 — Demographic data of the healthy controls. Abbreviations: MMSE = Mini Mental State Examination, MRI = Magnetic resonance imaging, n.d. = not done, UPDRS-III = Unified Parkinson’s Disease Rating Scale part III, FT = Finger tapping, le = left, ri = right, f = female, m = male, T initial = initial examination, T final = final examination. (DOC) [file pone.0041873.s001.doc]

| Control | 1 | 2 | 3 | 4 | 5 | 6 | 7 | 8 | 9 | 10 | 11 |
| --- | --- | --- | --- | --- | --- | --- | --- | --- | --- | --- | --- |
| Gender | f | f | f | m | f | f | m | m | m | m | f |
| Age, y | 65 | 68 | 60 | 79 | 60 | 66 | 45 | 65 | 45 | 50 | 58 |
| Education, years | >12 | <12 | <12 | >12 | <12 | <12 | <12 | <12 | >12 | >12 | <12 |
| Handedness | ri | ri | ri | ri | ri | ri | ri | ri | ri | ri | ri |
| Diagnostic MRI (T initial) | normal | normal | normal | normal | normal | normal | normal | normal | normal | normal | normal |
| MRI acquisition (Tesla) | 1.5 | 1.5 | 1.5 | 1.5 | 1.5 | 1.5 | 3.0 | 3.0 | 3.0 | 3.0 | 3.0 |
| Follow-up, months | 22 | 22 | 22 | 21 | 20 | 21 | 19 | 26 | 18 | 18 | 26 |
| *Motor testing* |  |  |  |  |  |  |  |  |  |  |  |
| UPDRS-III (T initial) | 0 | 2 | 0 | 3 | 0 | 0 | 0 | n.d. | 0 | 3 | n.d |
| UPDRS-III (T final) | 1 | 1 | 0 | 2 | 0 | 0 | 0 | 4 | 0 | 2 | 2 |
| *Neuropsychological testing* |  |  |  |  |  |  |  |  |  |  |  |
| MMSE (T initial) | 30 / 30 | 30 / 30 | 30 / 30 | 30 / 30 | 30 / 30 | 30 / 30 | n.d. | 29 / 30 | n.d. | 30 / 30 | 30 / 30 |
| MMSE (T final) | 30 / 30 | 30 / 30 | 30 / 30 | 30 / 30 | 30 / 30 | 30 / 30 | 30 / 30 | 29 / 30 | 30 / 30 | 30 / 30 | 30 / 30 |
